# ANALYSIS OF DIFFERENCE OF FOOT PLANTAR PRESSURE IN OLDER WOMEN WITH DIFFERENT BONE MINERAL DENSITY LEVELS

**DOI:** 10.1093/geroni/igae098.3403

**Published:** 2024-12-31

**Authors:** Min Liu, Gong Chen, Dongmin Wang, Ning Kang

**Affiliations:** Peking University, Beijing, Beijing, China (People’s Republic); Peking University, Beijing, Beijing, China; Peking University, Beijing, Beijing, China (People’s Republic); Peking University, Beijing, Beijing, China (People’s Republic)

## Abstract

**Objectives:**

To explore the characteristics of foot plantar pressures in the older women with different bone mineral density levels.

**Methods:**

50 older women (68.84 ± 3.73 yr) were recruited from one community in Beijing, China. T value of the bone mineral density and average force of 10 plantar partitions (toe 1, toe 2-5, metatarsal 1, metatarsal 2 and metatarsal 3, metatarsal 4, metatarsal 5, midfoot, medial heel, and lateral heel) in two feet were tested. Taking the median of T value, the participants were divided into low and high bone mineral density group (L group: N = 27, T = -3.43 ± 0.80, 68.62 ± 3.42 yr; H group: N = 23, T = -1.37 ± 0.68, 69.09 ± 4.12 yr). Independent T test was used to analyze the difference of foot plantar pressures between two groups.

**Results:**

(1) The average force of toe 1 on the dominant side in the L group was significantly lower than that in the H group (t = -2.161, p = 0.036). (2) The average force of metatarsal 5 on the dominant side and that of metatarsal 1 on the non-dominant side were significantly higher than that in the H group (t = 2.413, p = 0.020; t = 2.182, p = 0.034). Conclusions: The difference of average force in the older women with different bone mineral density levels was greater on the dominant side than that on the non-dominant side, and greater on the forefoot than that on the arch and heel.

